# Tragacanth Gum Hydrogel-Derived Trimetallic Nanoparticles Supported on Porous Carbon Catalyst for Urea Electrooxidation

**DOI:** 10.3390/gels8050292

**Published:** 2022-05-09

**Authors:** Badr M. Thamer, Meera Moydeen Abdulhameed, Mohamed H. El-Newehy

**Affiliations:** Department of Chemistry, College of Science, King Saud University, Riyadh 11451, Saudi Arabia; malhameed@ksu.edu.sa

**Keywords:** trimetallic nanoparticles, hydrogel, urea oxidation, fuel cells

## Abstract

The fabrication of electrocatalysts with high catalytic activity, high durability and low cost towards urea oxidation by a facile method is a great challenge. In this study, non-precious NiCoFe trimetallic supported on porous carbon (NiCoFe@PC) was prepared via gelation and pyrolysis method, presenting a remarkable electrocatalytic activity with low onset potential for urea oxidation in an alkaline medium. Field-emission scanning electron microscopy (FESEM) and transmission electron microscopy (TEM) were used to clarify the morphology of the NiCoFe@PC nanostructure and its nanoparticle size of 17.77 nm. The prepared catalyst with the composition ratio of 24.67, 5.92 and 5.11% for Ni, Fe and Co, respectively, with highly crystalline nanoparticles, fixed on porous carbon, according to energy-dispersive X-ray (EDX) and X-ray diffraction (XRD) analysis. The FeCoNi@PC catalyst showed a catalytic activity of 44.65 mA/cm^2^ at 0.57 V vs. Ag/AgCl and a low onset potential of 218 mV, which is superior to many other transition bi/trimetallic-based catalysts previously reported.

## 1. Introduction

The synthesis of electrocatalysts using facile methods based on available and low-cost materials is an important demand to reduce the cost and expand the use of alternative and sustainable energy devices. Fuel cells are one of the alternative energy sources to fossil fuels as they depend on renewable fuels and are less polluting to the environment such as hydrogen, methanol, ethanol and urea [1,2,3,4]. Recently, direct urea fuel cells (DUFCs) have sparked broad attention due to inherent characteristics of urea as fuel, such as high theoretical density (16.9 MJ/L), higher solubility (1079 g/L at 20 °C), availability, non-toxicity, non-flammability and simplicity of transport and storage [5]. Moreover, the use of urea as fuel will address the problem of pollution, as urea represents 2–2.5% of the total content of human and animal urine, making it a common pollutant for water and soil [6]. Despite the unique characteristics of urea as a fuel compared to methanol and hydrogen, the practical DUFCs still face many obstacles, including sluggish anode kinetics, declined power output, low durability and incomplete oxidation of urea [7]. The development of anode catalysts is the most significant pathway for enhancing the performance of practical DUFCs. Therefore, great efforts by researchers have been made to improve the performance of the anode starting from the utilization of catalysts based on precious metals such as Ti-Pt, Ti-Pt-Ir and Ru-TiO_2_ [8]. However, the electrocatalytic activity of precious catalysts was low in addition to their high cost. Then, as an alternative to precious catalysts, attempts were undertaken to fabricate catalysts based on non-precious metals and study their electrocatalytic activity towards urea oxidation [9,10]. Nickel-based catalysts are a good alternative due to their ability to establish bonds with urea molecules, which is associated with the interaction of unoccupied *d*-orbitals or unpaired *d*-electrons with the catalytically active Ni^3+^ state [11]. Moreover, nickel catalysts, especially at the nanoscale, have high extraordinary electrical conductivity (≈16 × 10^6^ S/m), high carrier concentration (≈3 × 10^18^ cm^−3^), wide bandgap (≈3.8 eV), availability of various oxidation states (Ni^0^/Ni^1+/^Ni^2+^/Ni^3+^) and high thermal stability and chemical stability [5]. Although nickel-based catalysts exhibited higher electrocatalytic activity than precious-metal-based catalysts, high onset potential, poor stability and surface poisoning effects hampered urea oxidation kinetics. These issues can be solved by merging two or more electron-rich metal atoms in a bi/trimetallic structure, which results in unique electronic and geometric effects as well as a better electrocatalytic activity than monometallic catalysts [12,13]. The incorporation of non-precious metals to Ni-based catalysts marks structural defects, which may improve the electrocatalytic activity and electronic conductivity, to dispense a larger active edge site for electrooxidation [14,15]. Electrocatalysts based on trimetallic alloy nanoparticles have better electrocatalytic activity and durability than bimetallic ones [16]. King et al., considered the effect of both cobalt and chromium electrodeposition onto nickel substrate on the catalytic activity, where they found that the Pt and Ir show an important role in the catalytic activity towards the oxidation of urea in the alkaline medium [17]. Botte and co-workers synthesized Ni-Zn-Co electrocatalyst by electrodeposition method and studied the effects of emerging Zn and Co metal on electrocatalytic activity of a Ni catalyst towards urea oxidation [18]. They found that the electrocatalytic activity of the Ni-Co-Zn catalyst was threefold greater than that of Ni itself, while the onset potential was reduced from 0.43 for Ni to 0.35 V for Ni-Co-Zn catalyst. Basumatary et al., investigated the effect of emerging Mn and Fe to a Ni catalyst as a tri-metallic catalyst on urea oxidation, and they found that the incorporation of both metals increased the catalytic activity by fourfold [19]. Generally, the design of trimetallic catalyst by the incorporation of non-precious metals such as Co, Fe, etc., with Ni is significant due to its abundance with low cost and tunable oxidation-dependent properties. This method is simple, clean and can be used in fabrication of new electrocatalysts for the electrooxidation of urea in an alkaline medium. Direct pyrolysis of carbon and metal precursor mixtures is the most common method for preparing trimetallic catalysts. However, the catalyst preparation process by this method is difficult to control, and metal precursors are disorderly scattered on, or randomly combined with, carbon precursors [20]. Hydrogel exhibits a unique morphology with hierarchical pores and contains multi-functional groups, which can be used as a template to fabricate numerous metal nanoparticles supported on carbon frameworks [21]. Tragacanth gum is a common nonlinear biopolymer containing oxygen functional groups. The abundance of negative-charge groups allows the creation of 3D cross-linked gels with positively charged metal ions via supramolecular self-assembly, which is used as a precursor for porous carbon. It is worthy to mention that tragacanth gum hydrogels have not been reported to fabricate bi/trimetallic electrocatalysts for urea electrooxidation.

In this study, we report the tragacanth gum hydrogel-based gelation/pyrolysis method to fabricate porous carbon-supported NiCoFe nanoparticles as trimetallic electrocatalysts. The as-prepared electrocatalyst was characterized by various techniques. The performances of the prepared electrocatalysts were investigated in terms of electrooxidation of urea by cyclic voltammetry (CV), chronoamperometry, and potentiometry techniques.

## 2. Experimental

### 2.1. Materials and Reagents

Tragacanth gum (TG) was bought from local market in Riyadh, Saudi Arabia. Nickel acetate tetrahydrate (NiAc), cobalt acetate tetrahydrate (CoAc), ferric chloride dihydrate (FeCl_3_), potassium hydroxide, urea, Nafion (5% *v*/*v*) and isopropanol were obtained from Sigma Aldrich Co., St. Louis, MO, USA.

### 2.2. Synthesis of NiCoFe@PC

**Scheme 1:**
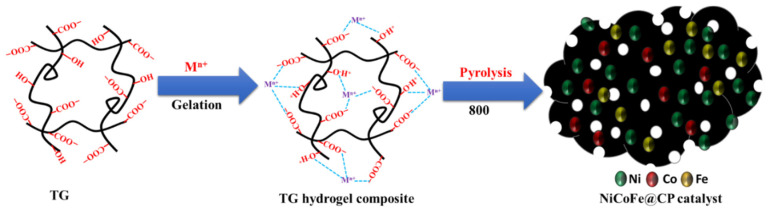
Schematic illustration of synthesis of NiCoFe@PC.

Initially, 1 g of TG (chemical structure is shown in Scheme S1) was dissolved in distilled water (50 mL) at 80 °C for 6 h. A 5.0 mL solution containing NiAc, CoAc, and FeCl_3_ at concentrations of 0.204 M, 0.068 M, and 0.083 M, respectively, was prepared. The metals solution was added to TG solution, followed by adding 0.5 g of urea at 60 °C until a brownish hydrogel was formed. Then, the hydrogel was cooled down and was dried by a vacuum oven at 25 °C for 72 h. Dried hydrogel composite was calcined in a tube furnace under nitrogen gas for 30 min at 200 °C and then 2 h at 800 °C, with a heating rate 5 °C/min in each step. Scheme 1 summarizes the preparation of catalyst steps based on gelation and pyrolysis processes.

### 2.3. Characterization of NiCoFe@PCs

The morphology and the particle size of the prepared catalyst were investigated by field-emission scanning electron microscopy (FESEM; EOL-JSM7100F, Tokyo, Japan) and transmission electron microscopy (TEM, JEOLJEM-2010, Tokyo, Japan). The chemical composition of the catalyst was investigated by X-ray diffraction (XRD; Bruker D8 DISCOVER, Berlin, Germany) and EDX analysis. Thermogravimetric analysis (TGA, Q500, TA, New Castle, DE, USA) was used to examine the best route of the hydrogel calcination process and carried out under nitrogen atmosphere.

### 2.4. Electrochemical Measurements

The VERSASTAT3 potentiostat was used for electrochemical measurements, with Pt wire as the counter electrode, Ag/AgCl (saturated KCl) as the reference electrode, and a coated glassy carbon electrode (GC; Diameter: 0.071 cm^2^) as the working electrode. The electrocatalytic activity and the stability of UOR were investigated using cyclic voltammetry (CV), chronopotentiometry (CP), and chronoamperometry (CA) experiments by VERSASTAT3. The working electrode was activated by carrying out 100 cycles from −0.2 V to 0.8 V with a scan rate of 50 mV/s in 1 M KOH before studying electrocatalytic activity and the stability. All CV and CA measurements were done at 296 ± 2 K, with the tested solutions exposed to the air. The working electrode was prepared as follows: 2.0 mg of the prepared catalysts was dispersed in 420 μL isopropanol by ultrasonication for 10 min. After that, Nafion (20 μL)was added to the dispersed catalyst and ultrasonication for 20 min. After that, the dispersed catalyst (15 μL) was dropped onto the surface of GC electrode in three batches.

## 3. Results and Discussion

### 3.1. Characterization of the Catalyst

#### 3.1.1. Morphology of NiCoFe@PC

**Figure 1:**
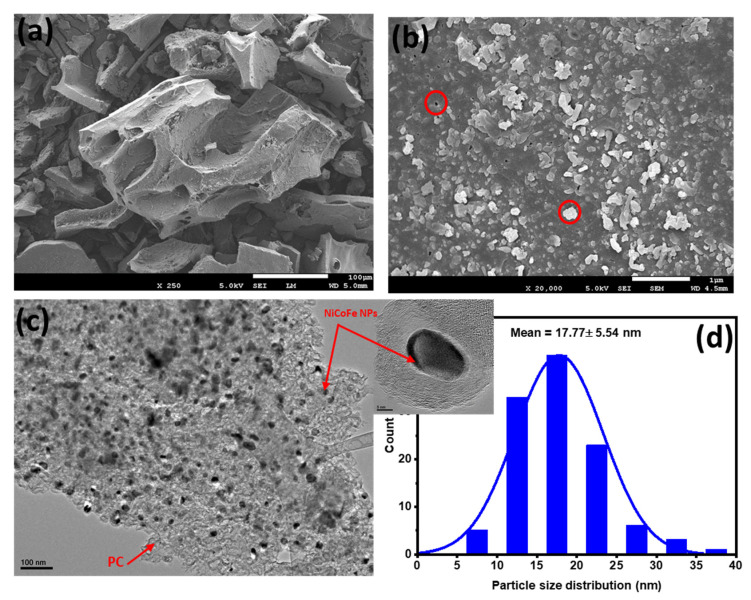
FESEM image of NiCoFe@PC; (**a**) low-magnification and (**b**) high-magnification, (**c**) TEM image of NiCoFe@PC of low and high resolution and (**d**) particle size distribution.

SEM, TEM, and HRTEM analysis were used to explore the morphology and the microstructure of NiCoFe@PC (Figure 1). Low-magnification FESEM image of the catalyst revealed an interesting 3D structure featuring rough surface and hierarchical open macrospores (Figure 1a). A closer inspection of the catalyst morphology in a high-magnification FESEM image revealed a mesoporous carbon structure composed of stacked NiCoFe nanoparticles (Figure 1b).

The morphology of the NiCoFe@PC was also explored by TEM analysis. As shown in Figure 1c, the NiCOFe NPs are uniformly distributed over the porous carbon surface with an average particle size of 17.77 ± 5.54 nm, which is consistent with XRD results.

#### 3.1.2. XRD and EDX Analysis of NiCoFe@PC

**Figure 2:**
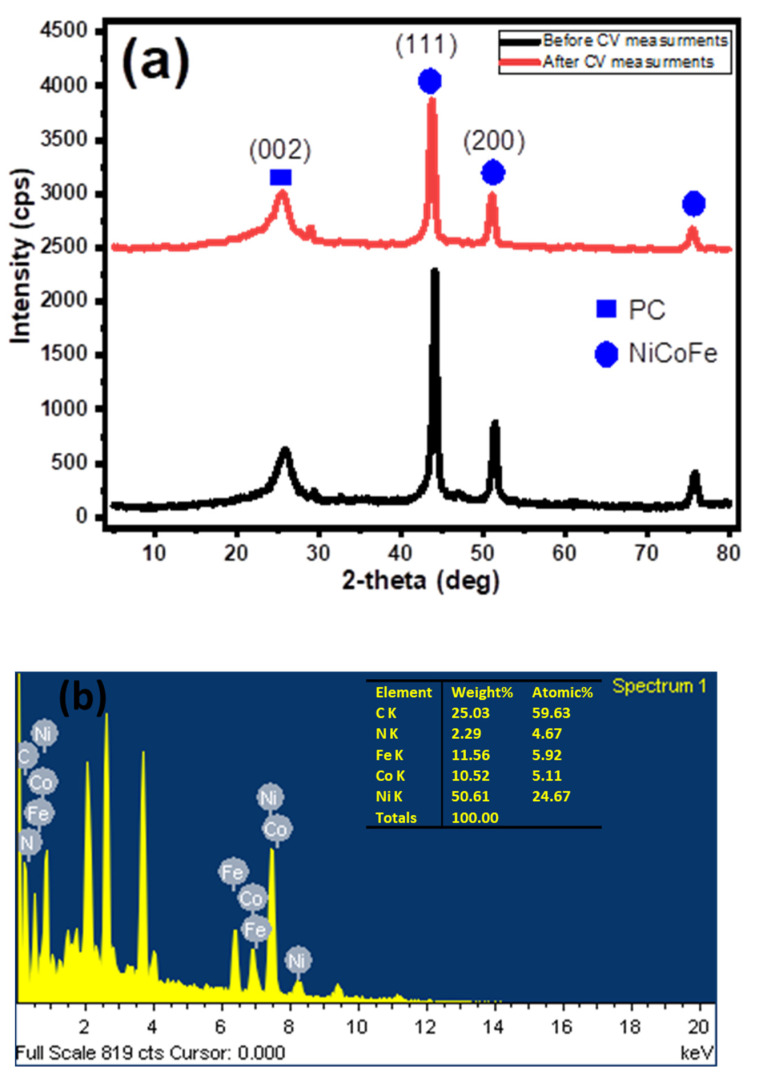
(**a**) XRD analysis of NiCoFe@PC before and after electrochemical measurements and (**b**) EDX analysis of NiCoFe@PC.

The XRD pattern of the NiCoFe@PC is displayed in Figure 2a. The sharp peaks centered at the 2*θ* values of 44.0, 51.5, and 75.8° are attributed to the face-centered cubic (fcc) phase crystallographic of trimetallic NiCoFe alloy, formed by the reduction during pyrolysis. These 2*θ* values agree with the findings in the literature [22,23]. The broad peak at 25.8° is allocated to the (002) plane of graphitic carbon, indicating an amorphous structure of the carbon matrix. Additionally, other weak pattern peaks at 29.4, 32.7 and 47° also appeared in the spectra, showing that the NiCoFe alloy sample is doped with a thin layer of metal oxide. Scherrer’s equation was used to calculate the average diameter of the crystallites and it was 13.37 nm based on the major three diffraction peaks’ analysis of fcc crystallographic structure of trimetallic NiCoFe alloy. After CV measurements and a long-term chronoamperometry test, the crystalline nature of NiCoFe@PC was further studied using XRD analysis. The XRD pattern of NiCoFe@PC, as shown in Figure 2a, demonstrates that only the peak intensity was lowered, with no changes in peak location or the appearance of additional peaks. This work exhibits good electrocatalyst stability of NiCoFe@PC in an alkaline medium during urea electrooxidation. The surface chemical composition of the NiCoFe@PC was investigated by EDX analysis (Figure 2b). EDX analysis revealed that the catalyst contains C, Ni, Co, Fe, and N atoms in the atomic ratio of 59.63, 24.67, 5.92, 5.11, and 4.67%, respectively, which is basically consistent with our preparation’s stoichiometry.

#### 3.1.3. Thermogravimetric Analysis of NiCoFe@PC

**Figure 3:**
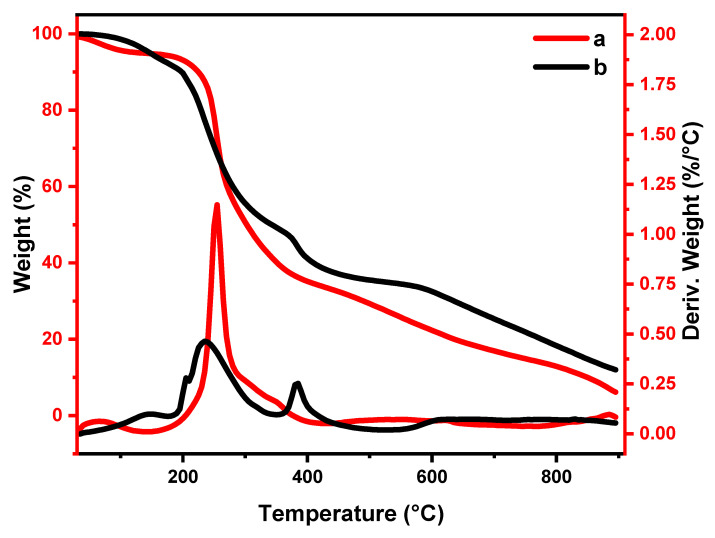
TGA/DTA analysis of (a) TG and (b) TG hydrogel trimetallic composite.

The thermogravimetric analysis of the pure TG, aerogel film is shown in Figure 3. The thermal degradation of TG took place in three steps. The first step indicated the evaporation of physically attached water to the TG that occurred at 50–120 °C. The second step is the main step, which occurred between 190 and 290 °C, and reflected the degradation of organic TG components. The third stage occurred at a temperature above 300 °C, indicating the degradation of the more stable residues. At a temperature of 800 °C, the residual weight percentage of TG was 12%. The thermal degradation of the TG/metal salts hydrogel took place in four steps. The first step at 40–180 °C was owing to the loss of physically attached water molecules. The second step represents the main degradation step for TG in the range between 190 and 350 °C in addition to the release of H_2_O, CO_2_, and CO gases. The third step, which took place between 350 and 510 °C, was assigned to the reduction of metal oxide to metal nanoparticles. The final step occurs in a wide range between 540 and 800 °C, which indicated the completion of the reduction of metal oxides and the deterioration of the carbon structure. At 800 °C, the residual weight percentage of TG/metal salts hydrogel was 18.23%, which is greater than that of pure TG.

Therefore, based on the result of the TGA analysis of the TG/metal hydrogel composite, the pyrolysis process was carried out at 800 °C to ensure the reduction of metal ions to metal nanoparticles loaded on the porous carbon.

### 3.2. Electrochemical Activity Study

**Figure 4:**
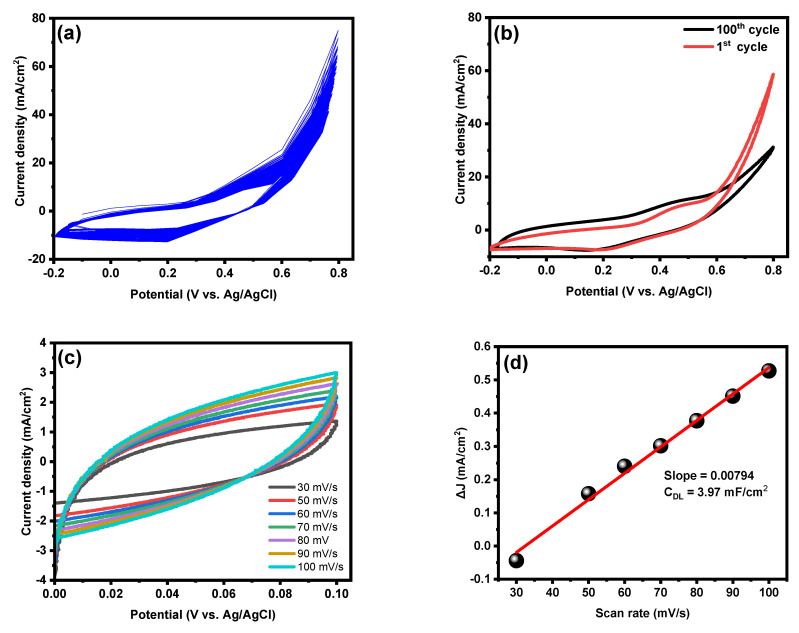
(**a**) 100 CV cycle in 1 M KOH with scan rate 50 mV/s (**b**) 1st and 100th cycle in 1 M KOH with scan rate 50 mV/s (**c**) CVs of prepared catalyst in a non-Faradaic region of voltammograms at scan rates of 30, 50, 60, 70, 80, 90 and 100 mV/s in 1 M KOH, and (**d**) matching linear fit of cathodic and anodic current density measured at 0.05 V vs. Ag/AgCl vs. scan rate.

Cyclic voltammetry tests in a three-electrode alkaline (1 M KOH) electrolyte were used to verify the electrocatalytic activity of NiCoFe@PCs. The activated catalyst was obtained before testing by sweeping it in a 1 M KOH solution for 100 cycles at a scan rate of 50 mV/s. The scan direction of the cycles started at −0.2 to 0.8 V (vs. Ag/AgCl) for the anode, then reversed from 0.8 to −0.2 V (vs. Ag/AgCl) for the cathode, as seen in the plots. Figure 4a shows the CV responses of NiCoFe@PC electrocatalyst in the blank 1 M KOH electrolyte at a scan rate of 50 mV/s. Two pairs of redox peaks in the potential range of 0.2 to 0.48 V can be seen in the behavior of the NiCoFe@PC, corresponding to (NiCoFe)(OH)_2_/(NiCoFe)OOH transformation. The creation of a (NiCOFe)(OH)_2_/(NiCoFe)OOH layer on the surface of the catalyst is a significant finding since it can be used as a sign of good electrocatalytic activity. For comparison, Figure 4b shows the CV curves for the 1st cycle and 100th cycle in a 1 M KOH electrolyte. After the 100th CV cycles, the cathodic peak at 0.21 V increased, as well as current density, which increased dramatically in the potential range of −0.2 to 0.6 V. This finding displayed that the active (NiCoFe)OOH species is required for UOR in an alkaline medium. The active surface area of the catalyst played a critical role in explaining the catalytic activity of the catalyst towards the oxidation of small molecules in an alkaline medium. To estimate the electrochemical active surface area (ECSA) that express the active sites of the catalyst, the difference in anodic and cathodic current density (ΔJ) at 0.05 V (vs. Ag/AgCl) was plotted vs. scan rates in the range of 30 to 100 mV/s to obtain a double-layer capacitance (C_DL_), as shown in Figure 4c,d. The NiCoFe@PC possessed the highest C_DL_ (3.97 mF/cm^2^), which is greater than the obtained values in previous study. The ECSA of the NiCoFe@PC can also be considered from the double-layer capacitance using the following equation:

$$ECSA=\frac{C_{DL}}{C_{s}}$$

where *C_s_* is the specific capacitance of the catalyst. Based on the previous studies, the value of the specific capacitance of various metals in the alkaline medium was reported in the range 0.022–0.130 mF/cm^2^. Therefore, the value of 0.04 mF/cm^2^ was used as general specific capacitance for the prepared catalyst [24]. Based on the values of *C_DL_* and *C_s_*, the ECSA value of the prepared catalyst was 99.25 cm^2^, which provided more active sites for the dynamic process of UOR.

**Figure 5:**
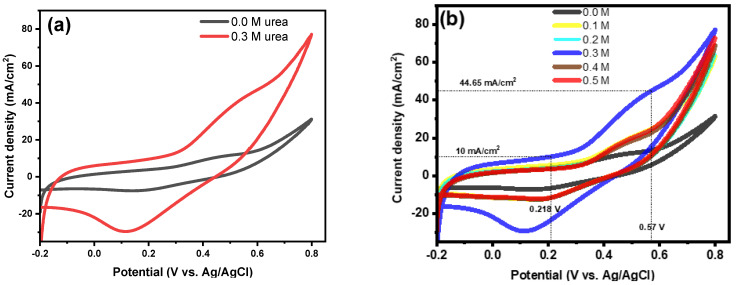

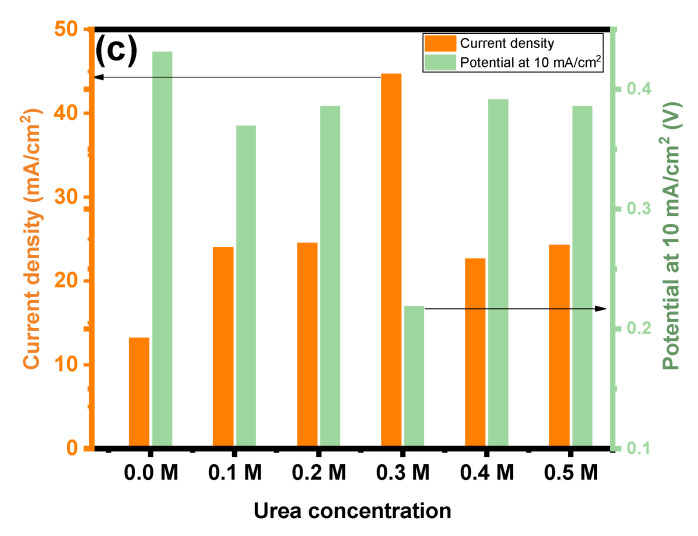
(**a**) CV measurements of NiCoFe@PC in 0.0 M and 0.3 M of urea, (**b**) CV measurements of NiCoFe@PC in different concentrations of urea and (**c**) plot of urea concentration vs. current density and potential at 10 mA/cm^2^. All measurements were carried out at 50 mV/s and 25 °C.

The CV measurements of NiCoFe@PC with and without urea are shown in Figure 5a. In 1 M KOH solution and 0.3 M urea, the anodic peak of current density increased largely and contrasting sharply in the absence of urea. Furthermore, the onset potential was found to be negative (−0.11 V) that is significantly lesser than the onset potential values for different electrocatalysts reported in previous studies as shown in Table 1. The current densities in 0.3 M urea are 5, 10, 45 and 77 mA/cm^2^ at a potential of −0.04, 0.22, 0.57 and 0.8 V, respectively. The concentration of urea has a high impact on the electrocatalytic activity of the catalyst. Figure 5b displays the effect of urea concentration on the electrocatalytic performance of NiCoFe@PC, as with increasing the urea concentration, the current density increased and reached its maximum value at urea concentration of 0.3 M, followed by a sharp decrease at 0.4 and 0.5 M concentrations. The increase in the current density at a concentration of less than 0.3 M can be ascribed to the fact that the active sites at lower concentrations are completely unoccupied and their number is higher than the reactant urea molecules, which results in faster reaction kinetics. However, after 0.3 M urea concentration, a slowdown in the reaction kinetics was observed, where at high concentrations, the number of reactant urea molecules is significantly higher than the number of unoccupied electroactive sites, resulting in an increase in the number of undecomposed intermediates that slow reaction kinetics [25,26,27]. It is worthy to note that the NiCoFe@PC exhibited a lesser onset potential in 0.3 M of urea, where the current density reached 10 mA/cm^2^ at 0.218 V, which is a more negative value than in the other concentrations as presented in Figure 5c. The onset potential of urea oxidation is consistent with the potential of NiOOH species creation. In the case of trimetallic NiCoFe NPs, the presence of urea (0.3 M), the high current density with redox peak appeared at 0.11 V consistent to the conversion of NiCoFe(OH)_2_ to NiCoFeOOH. Table 1 summarized a detailed comparison of electrocatalytic properties of the prepared catalyst and various catalysts based on precious bi/tri metallic for urea oxidation in an alkaline medium. The NiCoFe@PC catalyst showed high electrocatalytic activity as the required potential to flow 10 mA/cm^2^ was only 1.24 V vs. RHE. This value is among the lowest reported compared with NiCoP NS/CC [28], Ni_0_._85_Se/rGO [29], Ni-Cu/ZnO@MWCNT [30], NiZnCo [18], and NiSe/CNFs [31].

**Figure 6:**
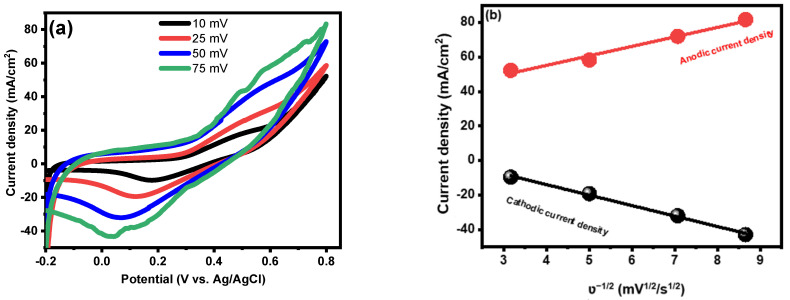

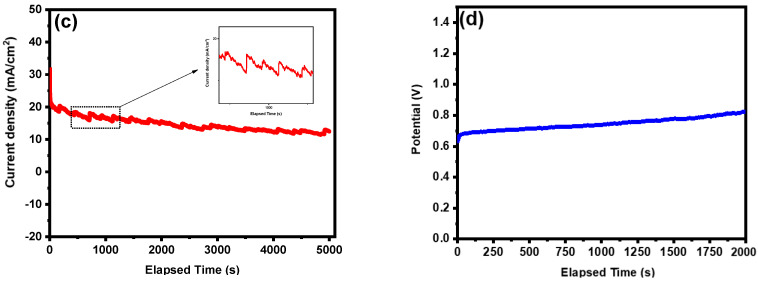
(**a**) CV measurements of NiCoFe@PC in 0.3 M of urea at different scan rate, (**b**) plotting of cathodic and anodic peak current densities vs. the square root of scan rate, (**c**) Chronoamperometric test in KOH solutions having 0.3 M urea at a potential value of 0.7 V for 5000 s and (**d**) chronopotentiometric test in 0.3 M urea at a current density value of 14 mA/cm^2^ for 2000 s.

The influence of the scan rate on the current density and the potential peak was used to investigate the electrochemical reaction mechanism of NiCoFe@PC catalyst towards urea oxidation. CV curves in 1 M KOH and 0.3 M urea at different scan speeds ranging from 10 to 75 mV/s are displayed in Figure 6a. Anodic peak current density increased linearly with the square root of the scan rate, as illustrated in Figure 6b. With regard to the Randles–Sevcik model, this result indicated that the urea electrooxidation on the NiCoFe@PC surface is a diffusion-controlled process.

The reverse-scanning peak current density was also plotted as a function of the square root of scan rate and exhibited a linear characteristic, indicating a reduction of NiCoFeOOH to NiCoFe(OH)_2_. This result indicated that the oxidation of NiCoFe(OH)_2_ to NiCoFeOOH in the anodic scanning takes place simultaneously with the oxidation of urea [35]. This result could be supported by plotting anodic current density × (scan rate)^−0.5^ (J × ν^−0.5^) versus scan rate (ν) as shown in Figure S1. This non-linear characteristic between J × ν^−0.5^ and ν confirmed also the occurrence of electrochemical oxidation of Ni(OH)_2_ into NiOOH and urea oxidation into CO_2_ and N_2_ [36].

The durability of the electrocatalyst is another criterion to test its efficiency towards urea oxidation in an alkaline medium. Chronoamperometry was used for further determination of the stability of NiCoFe@PC electrocatalyst for urea oxidation. Figure 6c displays the chronoamperograms of NiCoFe@PC performance in 1.0 M KOH solution having 0.3 M urea at a potential of +0.7 V for 5000 s. In the first seconds, the current density sharply decreased because the reaction was fast kinetic and active sites were free of adsorbed urea molecules, and then gradually decreased. Then, the adsorption of new urea molecules depends on the release of electrocatalytic sites by oxidation of urea or the intermediate species produced during the first few minutes (rate-limiting step) responsible for poisoning the active sites. As a result, the somewhat lower current density could be ascribed to the poisoning of the catalyst. However, the gradual decrease in the current density did not take place at a harmonious pace but fluctuated, which indicated that the catalyst got rid of the adsorbed intermediate species that poison its surface as shown in the inset Figure 6c. Due to the gradual decrease in the current density, the current density ratio retention at 1000, 2000, 3000, 4000, and 5000 s are 77.49, 72.29, 64.93, 57.62, and 58.43%, respectively. The anti-poisoning ability of NiCoFe@PC for urea oxidation was investigated by chronopotentiometry measurement in the solution of 0.3 M urea and 1 M KOH, as shown in Figure 6d. The chronopotentiometry measurement was performed at a current density of 14 mA/cm^2^ for 2000 s. It was observed from Figure 6d that the NiCoFe@PC demonstrated nearly constant operating potentials at 0.7 V vs. Ag/AgCl during 2000 s of urea oxidation testing. This result indicated that NiCoFe@PC can withstand poisoning intermediate species during 2000 s of urea oxidation, and it is consistent with XRD analysis after CV measurements and long-term stability test.

## 4. Conclusions

Gelation/pyrolysis as a facile and effective method was used to fabricate NiCoFe trimetallic NPs decorated by porous carbon using tragacanth gum and applied as a non-precious and effective catalyst for urea oxidation. According to the FESEM images, the NiCoFe@PC exhibited a metallic nanostructure supported on porous carbon. TEM analysis showed the metallic NPs were homogeneously distributed over the amorphous and porous carbon and had an average size of 17.77 nm. The NiCoFe@PC exhibited superior electrocatalytic activity (44.65 mA/cm^2^ at 0.57 V vs. Ag/AgCl) towards urea oxidation in alkaline media and significantly low onset oxidation potential (~281 mV vs. Ag/AgCl). Moreover, the potentiometric test showed that the NiCoFe@PC catalyst can resist poisoning within 2000 s of urea oxidation. The extreme simplicity and low cost of the gelation/pyrolysis-based method give it great promise for manufacturing electrocatalysts on an industrial scale.

## Figures and Tables

**Table 1:** Comparative electrocatalytic activity of numerous bi/trimetallic electrocatalysts for urea oxidation in alkaline media.

Catalyst	Concentration of KOH/Urea (M)	Potential at 10 mA/cm^2^ vs. RHE	Anodic Peak Current Density (mA/cm^2^) vs. RHE	Ref.
MnNiFe/rGO	1.0/0.33	-	2.86 at 1.523 V	[19]
NiCoP NS/CC	1.0/0.33	1.29	50 at 1.38	[28]
Ni_0_._85_ Se/rGO	1.0/0.5	1.36	-	[29]
NiCu/ZnO@MWCNT	0.4/0.07	1.36	30.02 at 1.38 V	[30]
CoNiCr/CNFs	1.0/0.33	-	21.34 at 1.523 V	[32]
NiZnCo	5.0/0.33	1.34	24 at 1.485	[18]
NiSe/CNFs	1.0/0.33	1.523	17 at 1.623 V	[31]
CNT/C@FeN	1.0/0.05	-	3.5 at 1.5 V	[33]
LaNiO_3_ Perovskite	1.0/0.33	-	28.58 at 1.48 V	[34]
NiCoFe@PC	1.0/0.3	1.24	44.65 at 1.593 V	This work

All the potentials reported in the literature and this study were adapted to RHE using the following equation: E_RHE_ = E_ref_ + 0.059 × pH + E^°^_ref_; where E_RHE_ is potential vs. reversible hydrogen electrode, E^°^_ref_ is standard potential of reference electrode vs. normal hydrogen electrode and E_ref_ is measured potential of reference electrode.

## Data Availability

Not Applicable.

## Supplementary Materials

The following are available online at https://www.mdpi.com/article/10.3390/gels8050292/s1, Scheme S1. Tragacanth gum struracture (a) xylogalacturonan unit and (b) arabinogalactan unit; Figure S1. plotting anodic current density × (scan rate)^−0.5^ (J × ν^−0.5^) versus scan rate (ν).
