# Supplementary material for: Tragacanth Gum Hydrogel-Derived Trimetallic Nanoparticles Supported on Porous Carbon Catalyst for Urea Electrooxidation
Source: Gels. 2022 May 9;8(5):292. doi: 10.3390/gels8050292 (PMC9141339; doi:10.3390/gels8050292)
Supplement: Supplementary file 1 [file gels-08-00292-s001.zip › gels-1694008-SI.pdf]

gels

MDPI

Article

Tragacanth Gum Hydrogel-Derived Trimetallic Nanoparticles Supported on Porous Carbon Catalyst for Urea Electrooxidation

Badr M. Thamer *, Meera Moydeen Abdulhameed, Mohamed H. El-Newehy *

Department of Chemistry, College of Science, King Saud University, Riyadh 11451, Saudi Arabia; malhameed@ksu.edu.sa

* Correspondence: bthamer@ksu.edu.sa (B.M.T.); melnewehy@ksu.edu.sa (M.H.E.-N.)

(a)

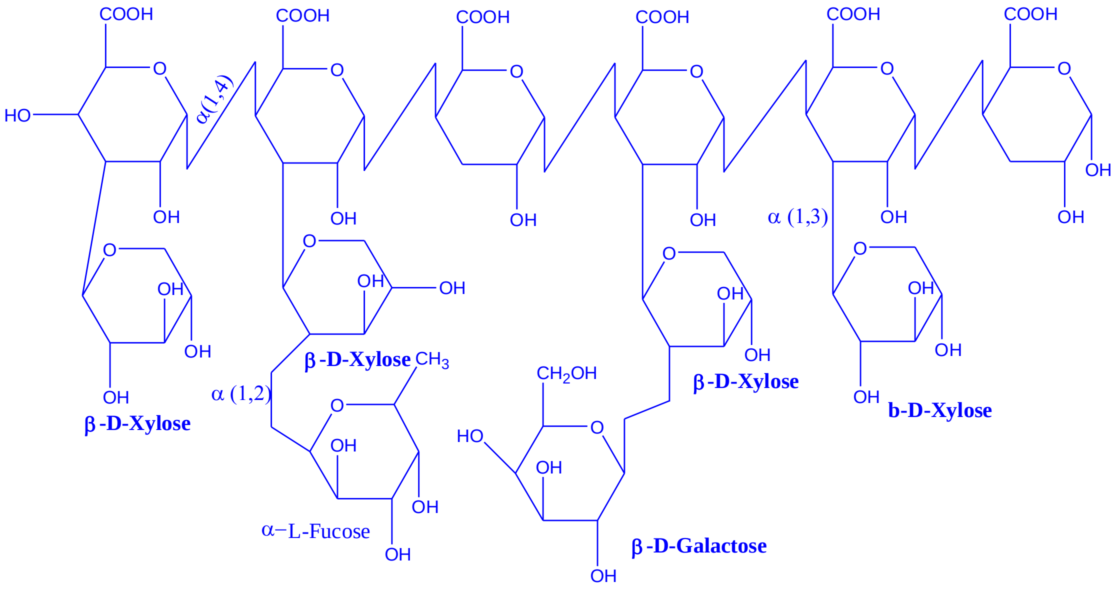

(b)

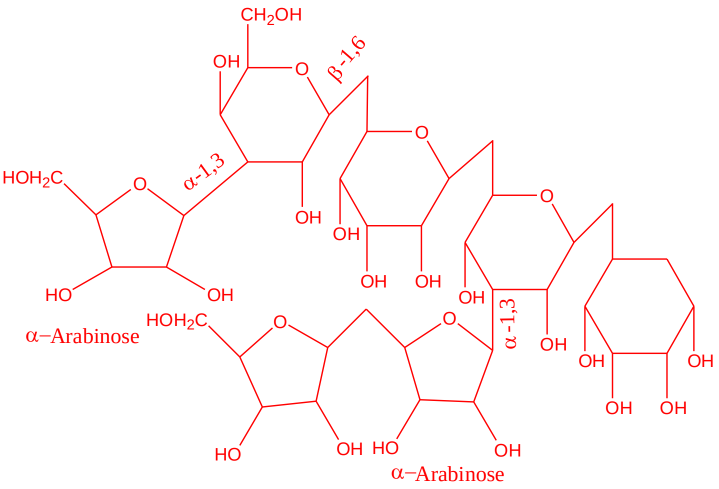

Scheme S1. Tragacanth gum struracture (**a**) xylogalacturonan unit and (**b**) arabinogalactan unit.

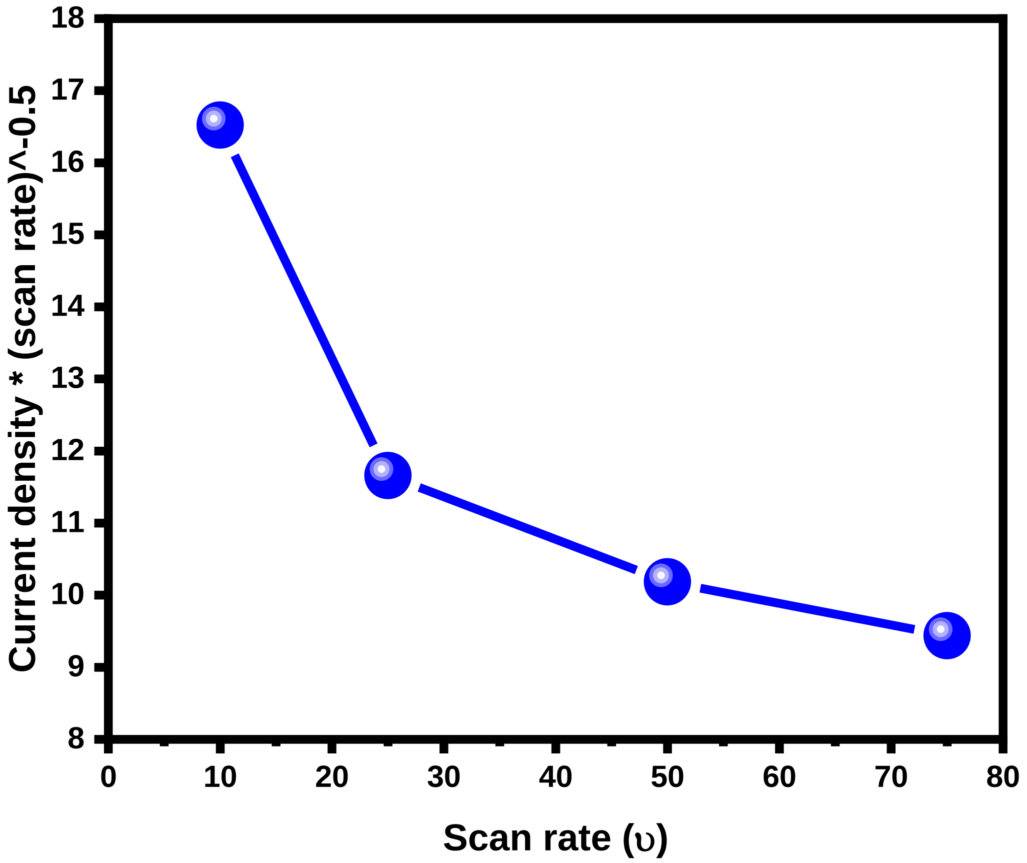

Figure S1. plotting anodic current density × (scan rate)$^{-0.5}$ ($J \times \nu^{-0.5}$) versus scan rate (ν).
